# An outbreak of hemolytic uremic syndrome due to Escherichia coli O157:H7: or was it?

**DOI:** 10.3201/eid0202.960218

**Published:** 1996

**Authors:** P N Goldwater, K A Bettelheim


### An Outbreak of Hemolytic Uremic Syndrome due to *Escherichia coli* O157:H-: Or Was It?

**To the Editor:** Since the first reported outbreaks of hemolytic uremic syndrome (HUS) and related conditions more than 10 years ago (1), outbreaks of HUS due to *Escherichia coli* O157 have been reported from many parts of the world, particularly North America and Europe. While most of these reports have incriminated the motile strains of serotype O157:H7, nonmotile serotypes (e.g., O157:H-) have also been associated with HUS; these two serotypes are most commonly associated with both outbreaks and sporadic cases of HUS and related conditions. Over the last decade, a number of techniques for the rapid identification of these organisms have been developed. Of these, the use of sorbitol-MacConkey agar (2) has perhaps been the most valuable. This technique is based on the fact that these organisms rarely ferment sorbitol on primary isolation, while most other *E. coli* usually ferment this substrate. We believe that outbreaks due to other enterohemorrhagic *E. coli* may have been attributed to serogroup O157 because of the limited technology used in investigating these outbreaks.

No outbreaks of HUS due to serogroup O157 have occurred in Australia despite sporadic cases of HUS caused by such strains. Other serogroups (particularly serotype O111:H-) have been associated with most cases of HUS and related conditions in Australia (3). No outbreak of HUS had been reported in Australia until January 1995, when an outbreak associated with the consumption of contaminated mettwurst (fermented sausage) was reported from South Australia (4). Twenty-three children with HUS were hospitalized. Most required hemodialysis; one died. Verocytotoxigenic strains of *E. coli* O111 producing Shiga-like toxin (SLT) I and II were isolated from 19 patients and from samples of mettwurst. In addition, strains of *E. coli* O157:H- that produced SLT-I and SLT-II were isolated from three of the patients and the mettwurst. These strains did not ferment sorbitol on the sorbitol-MacConkey agar, which facilitated their isolation. The predominant O111 strains were sorbitol-positive, unlike the O111 strains, recently described as being sorbitol-negative (5). Symptoms of the patients from whom the O157:H- strains were isolated, in addition to *E. coli* O111:H-, were not significantly different from those of the patients whose specimens yielded only *E. coli* O111:H-. In addition to O111 (and O157), other serotypes of enterohemorrhagic *E. coli*, including strains of serogroup O23, O26, and O91, were isolated from the patients. However, antibodies to O111 were detected in nearly all patients, which indicates the serogroup's leading role in the outbreak. The isolation of serogroup O157 is comparatively easy; therefore, it is less likely that these strains would have been missed, than it is that O111 and other serotypes would have been. Even though a negative finding can never be considered conclusive, we consider the inability to isolate serogroup O157 more conclusive than the same result for other serotypes. It has frequently been suggested that the O157 serogroup is cleared from the patient relatively rapidly, which makes its isolation difficult or impossible. We found a similar situation with other enterohemorrhagic *E. coli* serotypes. The fact that most patients elicited an O111 antibody response (and no anti-O157) almost certainly proves this serotype's causal role in this outbreak.

The laboratory in South Australia was particularly well disposed to deal with such an outbreak because some of its ongoing research programs included studies on aspects of enterohemorrhagic *E. coli* and related organisms. The most sophisticated molecular biologic techniques were immediately available to investigate the outbreak accurately and confirm epidemiologic leads regarding a common source. Polymerase chain reaction (PCR) played a major role not only in identifying SLT-I, SLT-II, and SLT-I and SLT-II producing bacteria in the stool of patients, but also in identifying the suspected source (mettwurst). In addition, PCR, utilizing sequences specific for the O111 serogroup, enabled this serogroup to be rapidly identified in patients' feces samples and suspected source material. Without this technology, the outbreak would not have been contained so rapidly. On the other hand, if the laboratory had to rely on conventional microbiologic culture procedures, including sorbitol-MacConkey agar, strains of serogroup O157 would have been identified from three patients, as well as from the epidemiologically incriminated mettwurst. The laboratory would not have found the O111 strains because they all fermented sorbitol readily and would have been discarded as normal flora as would the other enterohemorrhagic *E. coli* serotypes. The outbreak would have been reported as another O157 outbreak, from which only about 15% of the patients yielded the incriminating strains. This outbreak could be recognized as one caused by a number of different enterohemorrhagic *E. coli* serotypes, of which serotypes O111:H- and O157:H- were the most prominent.

Other serotypes, however, such as O23, O26, and O91, were also present. With the widespread nature of verocytotoxigenic strain of different serotypes as has been reported from many environmental studies, it is not surprising that a product, such as mettwurst, which is made from meats from various sources, would contain a number of these potential pathogens.

A large number of E. *coli* serotypes can be verocytotoxigenic and, in a few cases, outbreaks due to such strains have been reported. Most notable have been reports from Italy of outbreaks due to enterohemorrhagic *E. coli* O111 (6); however, the impression is that these are the exception and that the most prominent serotype is O157:H7. Some of the reported outbreaks due to O157 strains may in fact have been due to other serotypes and the O157 strains were only present in comparatively small numbers; however, because of the ease with which these strains can be identified using sorbitol-MacConkey agar, they were believed responsible for the outbreaks. For example, in Argentina, *E. coli* O157:H7 was found in only one (2%) of 51 children with HUS (7) and in the Netherlands, only 5 (19%) of 26 HUS patients yielded *E. coli* O157:H7 (8). In a 10-year, retrospective, population-based study of HUS, this serotype was isolated in 13 (46%) of 28 patients (9), and in their review, Su and Brandt (10) put an overall figure of 46% to 58% as the incidence range of E. *coli* O157:H7 infection in cases of HUS. Finding SLT sequences in a fecal specimen by PCR, or free fecal toxins in many patients of an outbreak while isolating strains of O157 from only a few, does not exclude the presence of other serotypes, but culture methods now available would rarely pick these up. Thus there is ample room to speculate that approximately half the cases of HUS may be caused by serogroups other than O157 and, by inference, at least half the outbreaks may be wrongly attributed to this serogroup. We recognize that enterohemorrhagic *E. coli* O157 have become extraordinarily widespread throughout the world since their first description (1); this does not mean that other serotypes are not also causing infections, either alone, in conjunction with O157, or even with other known or unknown enteric infections. It is important to be aware of the existence of these other serotypes and be vigilant for them. The isolation and characterization of strains of serogroup O157 from patients with HUS is certainly noteworthy, but so is the finding of O111 or any other serogroup. Serogroup O111 has amply demonstrated the ability to cause extensive outbreaks (6). Even though many laboratories are becoming aware of the importance of testing for serogroup O157:H7, we think that testing for this serotype only is a disservice; simple culture techniques can identify this serogroup, but always at the risk of missing other serogroups. The development of simple methods to detect all enterohemorrhagic *E. coli* is now required.

**P.N. Goldwater, F.R.A.C.P., F.R.C.P.A.*, and K.A. Bettelheim, Ph.D.†**
*Women's and Children's Hospital, Adelaide, South Australia; †Biomedical Reference Laboratory, Victorian Infectious Diseases Reference Laboratory, Fairfield Hospital, Victoria, Australia

### References

1. Riley LW, Remis RS, Helgerson SD, McGee HB, et al. Hemorrhagic colitis associated with a rare *Escherichia coli* serotype. N Engl J Med 1983;308:681-5.
2. March SB, Ratnam S. Sorbitol-MacConkey medium for detection of *Escherichia coli* O157.H7 associated with hemorrhagic colitis. J Clin Microbiol 1986;23:869-72.
3. Goldwater PN, Bettelheim KA. The role of enterohemorrhagic *Escherichia coli* serotypes other than O157:H7 as causes of disease. Communicable Disease Intelligence 1995;19:2-4.
4. Cameron S, Walker C, Beers M, Rose N, and Anear E, et al. Enterohemorrhagic *Escherichia coli* outbreak in South Australia associated with consumption of mettwurst. Communicable Disease Intelligence 1995;19:70-1.
5. Ojeda, A, Prado, V, Martinez, J, et al. Sorbitol-negative phenotype among enterohemorrhagic *Escherichia coli* strains of different serotypes and from different sources. J Clin Microbiol 1995;33:2199-201.
6. Caprioli A, Luzzi I, Rosmini F, Resti C, et al. Communitywide outbreak of hemolytic-uremic syndrome associated with non-O157 verocytotoxin-producing *Escherichia coli*. J Infect Dis 1994;169:208-11.
7. Lopez EL, Diaz M, Grinstein S, Devoto S, et al. Hemolytic uremic syndrome and diarrhea in Argentine children: the role of Shiga-like toxins. J Infect Dis 1989;160:469-75.
8. van der Kar NCAJ, Roelofs HGR, Muytjens HL, et al. Verocytotoxin-producing *Escherichia coli* infection in patients with hemolytic uremic syndrome and their family members in the Netherlands. In: Kaarmali MA, Goglio AG, editors. Recent advances in verocytotoxin-producing *Escherichia coli* infections. Amsterdam: Elsevier, 1994.
9. Martin DL, MacDonald KL, White KE, Soler JT, Osterholm MT. The epidemiology and clinical aspects of the hemolytic uremic syndrome in Minnesota. N Engl J Med 1990;323:1161-7.
10. Su C, Brandt LJ. *Escherichia coli* O157:H7 infections in humans. Ann Intern Med 1995;123:698-714.

